# Prevalence and sociodemographic factors associated with meeting the 24-hour movement guidelines in a sample of Brazilian adolescents

**DOI:** 10.1371/journal.pone.0239833

**Published:** 2020-09-28

**Authors:** Bruno G. G. da Costa, Jean-Philippe Chaput, Marcus V. V. Lopes, Luís E. A. Malheiros, Mark S. Tremblay, Kelly S. Silva

**Affiliations:** 1 Research Group in Physical Activity and Health, Universidade Federal de Santa Catarina, Florianópolis, Brazil; 2 Healthy Active Living and Obesity Research Group, Children’s Hospital of Eastern Ontario Research Institute, Ottawa, Ontario, Canada; University of Idaho, UNITED STATES

## Abstract

**Background:**

The present cross-sectional study aimed to determine the proportion of adolescents meeting the 24-hour movement guidelines, and investigate sociodemographic factors associated with meeting them.

**Methods:**

Self-reported (average daily volume of MVPA, sleep duration, and time watching videos and playing videogames) and accelerometer-measured (MVPA and sleep duration) 24-hour movement behaviors were classified according to recommendations, and sex, age, socioeconomic status (SES), family structure, parental education, and number of people in the household were tested as correlates of meeting recommendations using multilevel logistic regressions.

**Results:**

The proportion of adolescents (n = 867, mean age: 16.4 years, 50.3% girls) meeting the MVPA, ST, and sleep duration guidelines was of 25%, 28%, and 41%, respectively, for self-reported data. From accelerometer data (n = 688), 7.1% met MVPA and 31.7% met sleep duration recommendations. Adherence to all three recommendations was 3% with self-report and 0.2% with accelerometer data. Boys were more likely to meet MVPA, but not ST and sleep-duration recommendations. A positive relationship was observed between age and meeting the ST recommendation.

**Conclusions:**

Adherence to the sleep duration recommendation was higher than to the screen-time and MVPA recommendations and few in this sample of Brazilian adolescents achieved the 24-hour guidelines. Efforts are needed to improve 24-hour movement behaviors.

## Introduction

Physical activity, sedentary behavior, and sleep are important behaviors that are each associated with cardiometabolic and mental health of children and adolescents [1–5]. The benefits of practicing regular physical activity, preventing excessive sedentary behavior, and having adequate sleep have resulted in the publication of specific guidelines for each of these behaviors after reviewing the best available evidence [3,6–8,9(p20)]. The world’s first 24-hour movement guidelines that integrate physical activity, sedentary, and sleep behavior recommendations were published in 2016 by Canada [10], and soon after by New Zealand, Australia [11], and others [1]. These guidelines suggest that adolescents should engage in 60 minutes of moderate-and-vigorous intensity physical activity (MVPA) daily [10–12], should avoid spending more than 2 hours per day in leisure-time screen activities [10,11] (such as watching television or playing videogames), and should accumulate enough sleep (8–10 hours per night for adolescents between 14 and 17 years) [10]. However, when assessing behaviors according to these criteria, many adolescents do not engage in enough MVPA [13,14], spend excessive time on screen-based activities [14], and do not obtain sufficient sleep [15,16].

Adhering to all guidelines is associated with more health benefits compared to meeting just one or none [17–19]; however, adherence to 24-hour movement guidelines is low. Only 7.2% of 9–11 year-olds were found to meet all three recommendations in a study with 12 countries between 2011 and 2013 [18(p)], while 3% met all recommendations in a study with 22,115 Canadian adolescents (10–17 years) in 2016 [20], and 5% of adolescents from the United States [21]. Furthermore, some population subgroups may be at increased risk of not meeting one [22] or all guideline recommendations. For example, boys commonly engage in more MVPA [22] and sleep better [23–26] when compared to girls. Age is inversely associated with physical activity [22] and sleep duration [26] through adolescence, while an increase in recreational screen time is observed [27]. Lower socioeconomic status (SES) is also associated with less physical activity [22], less screentime [27], and poorer sleep [28,29]. And recently, the family structure (e.g., living with both parents or single parent) has also been shown to be associated with screen time [30]. Fewer girls and fewer older adolescents seem to meet all 24-hour movement recommendations compared to boys and younger peers in the United States [21], while age was also inversely related to meeting all the guidelines simultaneously among Canadian adolescents [20].

Evidence on correlates of the 24-hour movement behaviors are from studies conducted predominantly on high-income countries [20–22], and may not reflect behaviors in middle- and low-income countries. This difference can be illustrated by the relationship with SES, that may be positively or negatively related with MVPA [22] or screen time [31] depending on the level of country development [32]. The aims of the present study were to i) determine the proportion of adolescents from Florianopolis, Brazil meeting the 24-hour movement guidelines; and ii) investigate sociodemographic factors associated with meeting the 24-hour movement guidelines.

## Material and methods

### Population and sample

Cross-sectional data from the baseline sample of the *Estudo Longitudinal do Estilo de Vida de Adolescentes* (ELEVA: Longitudinal study of the lifestyle of adolescents) was used. All adolescents (age range: 14–18 years) enrolled in public high schools integrated with professional courses of all schools in the mesoregion of Florianópolis, southern Brazil, were invited to participate. Data collection was conducted between August and December 2019. A total of 1618 students were listed by the schools (n = 3), and 1269 were at the school during data collection visits and were invited to participate. Informed consent forms were given to students for their parents or legal guardians to sign, and an assent form was given to all students to sign. A total of 1010 students and parents signed the forms and were thus able to take part in the study. The research project was approved by the Ethics Committee in Research with Human Beings of the *Universidade Federal de Santa Catarina* (protocol number: 3.168.745).

### Measures

Physical activity and sleep duration were measured using Actigraph GT3x+ and wGT3x+ accelerometers. Trained researchers instructed the participants to wear the accelerometer 24-hours per day for one week on the non-dominant wrist, secured by a disposable PVC band. Participants were oriented to remove the accelerometer during water activities if the monitor would be submerged (e.g., surfing, swimming), but not for other water-based activities such as showering or washing the dishes. Accelerometers were programmed using a 30 Hz sampling frequency, and data were analyzed using 5-second *epochs*. Valid wear-time was identified using an adapted version of the analysis described by Van Hees et al. [33]. This algorithm looks at blocks of 15 minutes within each 60 minute windows, and classifies each block as non-wear if the standard deviation of the 60-minute window is less than 13.0 m*g* (1m*g* = 0.00981m/s^2^) for at least two out of the three axes or if the value range, for at least two out of three axes, was less than 50 m*g*. Participants were included in the analyses if they provided 4 days with 16 hours of valid accelerometer data after exclusion of non-wear time. Acceleration of the vector magnitude was analyzed, and activities above 201.4 mg per epoch were classified as MVPA [34]. Sleep duration was derived from the accelerometry data using the Heuristic algorithm looking at Distribution of Change in Z-Angle presented by Van Hees [35]. The sleep duration variable was estimated using the difference between sleep onset and wake-up, without excluding awakening periods. Analyses of raw accelerometer data were conducted using the GGIR package [36].

Self-reported physical activity was measured using the *Self-Assessed Physical Activity Questionnaire*, where participants reported weekly frequency (number of days/week) and duration (minutes/day) they practiced physical activity from a list of 22 activities, with a space for adding non-listed activities. This instrument has been validated for Brazilian adolescents [37]. The weekly volume of MVPA was calculated by summing the volume (frequency*duration) of the listed activities, and the cut-off of 420 minutes/week was used as equivalent to 60 minutes/day according to the guidelines for this age group.

Screen time was assessed using two questions where participants answered the total hours and minutes per weekday or weekend day that they spend watching videos (e.g., movies, series, news) and playing videogames. The questions stated that they should consider time spent watching videos or playing on a smartphone, television, computer, tablet or any other electronic device. The answers were weighted using the following formula: ([volume on weekdays*5 + volume on weekend days*2]/7), and classified using the cut-off point of 2 h/day. This procedure was validated in a previous pilot study (n = 104 adolescents), yielding Gwet’s agreement coefficient of 0.79.

Sleep duration was estimated using the difference between the self-reported sleep onset (hour and minutes) and wake-up during weekdays. This question has been used in previous studies with Brazilian adolescents [38,39]. Implausible sleep duration values (<1 or >20, n = 3) were interpreted as errors and dropped. Sleep duration was classified into meeting vs. not meeting the recommendations of 8–10 hours of sleep per night, as recommended for this age group [10].

Sociodemographic variables included sex, age, SES, parental education, family structure (i.e., live with both parents, live with mother, live with father, live without father and mother), and number of people living in the same household. SES was calculated using a score derived from ownership of the following household items: bathrooms, housemaids, cars, computers, dish washers, fridges, freezers, washing machines, DVD players, microwave ovens, motorcycles, drying machines; highest education level of the family; having white water, and living on a paved street. The weight of each item was according to the Brazilian Association of Research Companies [40].

### Statistical analyses

Participants’ characteristics were described using means and standard deviations, and relative and absolute frequencies for continuous and categorical variables, respectively. Comparison of characteristics between those who provided valid accelerometer data and those who did not was conducted using Student’*s t-*tests and Pearson’s Chi Squared tests.

Mixed-effects logistic regression analyses were fit using adherence of all and each 24-hour movement behavior variables as dependent variables, and sociodemographic variables as independent variables considering the hierarchical structure of the data, where adolescents were nested within schools. Categorical variables were sex, family structure, and parental education, whereas age (in years), SES (in 0–100 score), and number of people in the household (count of people) were continuous. Analyses were conducted with R, version 3.6.0 for Windows, using the *lme4* package. Significance was set at p<0.05 (two-tailed).

## Results

Of the total of 1010 participants who provided consent and were authorized to participate, 837 (82.9%) provided responses for all questionnaire variables included in the present analyzes, and 688 (68.1%) provided valid accelerometer data on 4 or more days. Participants were 16.4±1.1 years old, and half (50.3%) were girls. Most participants (63.2%) lived with both parents, and had at least one parent with more than 11 years of education (60.1%). The sleep duration had the highest proportion of meeting the recommendation (41%), followed by screen time (28.6%) and MVPA (25.1%), when self-report data were analyzed. When accelerometer data were analyzed, 7% met the MVPA guideline recommendation while 31.6% met the sleep duration recommendation (Table 1). No differences were found by sex, SES, family structure, parental education, self-reported sedentary behavior, and self-reported sleep duration between those who provided valid accelerometer data and those who did not. However, the proportion of physically active participants with self-reported data was higher (34.9% *vs* 23.0%, p<0.05), and they were on average 0.36 years older (p<0.05, S1 Table).

**Table 1:** Descriptive characteristics of the sample.

		n (%) / Mean ± SD
**Sex**		
	Girls	421 (50.3)
	Boys	416 (49.7)
Age (years)*		16.4 ±1.1
SES score [0–100]*		48.9 ±10.0
Number of people in the household*		3.8 ±1.2
**Family structure**		
	Live with both parents	529 (63.2)
	Single parent	267 (31.9)
	Does not live with parents	41 (4.9)
**Highest education among parents**		
	>11 years	503 (60.1)
	8–11 years	275 (32.9)
	<8 years	44 (5.3)
	Unknown	15 (1.8)
***Recommendations using self-report***		
	MVPA (≥60 minutes/day)	210 (25.1)
	Screen time (≤2 hours/day)	239 (28.6)
	Sleep duration (8–10 hours/night)	345 (41.2)
**Recommendations using accelerometer data (n = 688)**		
	MVPA (≥ 60minutes/day)	49 (7.1)
	Sleep duration (8–10 hours/night)	218 (31.7)

*Mean and standard deviation (SD).

MVPA: Moderate-to-vigorous physical activity; SES: Socioeconomic status.

The proportion of participants meeting each combination of the 24-hour movement guidelines is presented in Table 2. When self-report data were analyzed, one third of students did not meet any of the guidelines, 42.3% met one recommendation, 21.6% met two recommendations, and only 3.1% met all recommendations. When MVPA and sleep duration were measured using accelerometers, 45.5% of the participants did not meet any of the guidelines, 41.6% met one (20.5% met sleep duration only), 12.8% met two (9.5% met sleep duration and screen time only), and 0.2% met all three recommendations.

**Table 2:** Proportion of participants meeting the MVPA, screen time, and sleep duration recommendations and various combinations based on self-report and accelerometer data.

	Self-report (n = 837)	Accelerometer-measured PA & Sleep (n = 688)
*Recommendations*	n (%)	n (%)
*All three*	26 (3.11)	1 (0.15)
*Two Only*		
Sleep duration & SB only	77 (9.2)	65 (9.45)
Sleep duration & PA only	68 (8.12)	11 (1.6)
PA & SB only	36 (4.3)	12 (1.74)
*One only*		
Sleep duration only	174 (20.79)	141 (20.49)
PA only	80 (9.56)	25 (3.63)
SB only	100 (11.95)	120 (17.44)
*None*	276 (32.97)	313 (45.49)

PA: Physical activity; SB: Sedentary behavior.

The association between sociodemographic indicators and meeting all three and each of the 24-hour movement guideline recommendations measured with self-report data can be observed in Table 3. Boys were more likely to meet MVPA guidelines (OR = 1.75, 95% CI: 1.28–2.40) compared to girls, but less likely to meet the screen time guidelines (OR = 0.40, 95% CI: 0.29–0.55). A positive relationship was found for age and odds of meeting the screen time guidelines (OR = 1.15, 95% CI: 1.00–1.32). No statistically significant associations were observed for the other sociodemographic indicators and meeting any or all of the 24-hour movement behavior guidelines.

**Table 3:** Sociodemographic correlates of meeting each and all of the 24-hour movement behavior guidelines using self-reported data (n = 837 adolescents).

		All		MVPA		Screen time		Sleep duration	
		OR	95%CI	OR	95%CI	OR	95%CI	OR	95%CI
**Sex**									
	Girls (ref)	1	1	1	1	1	1	1	1
	Boys	0.72	(0.32–1.61)	**1.75**	**(1.28–2.40)**	**0.40**	**(0.29–0.55)**	1.04	(0.79–1.37)
**Age (years)**		0.89	(0.62–1.26)	1.01	(0.88–1.16)	**1.15**	**(1.01–1.32)**	0.89	(0.77–1.02)
**SES score**		1.02	(0.98–1.06)	1.01	(0.99–1.03)	0.99	(0.97–1.01)	1.00	(0.98–1.02)
**Number of people in the household**		1.00	(0.70–1.42)	1.05	(0.92–1.21)	1.09	(0.95–1.26)	0.96	(0.85–1.08)
**Family structure**									
	Live with both parents (ref)	1	1	1	1	1	1	1	1
	Single-parent	0.79	(0.32–2.00)	1.13	(0.78–1.64)	0.86	(0.60–1.22)	0.86	(0.62–1.20)
	Does not live with parents	x	x	0.75	(0.33–1.70)	0.84	(0.40–1.78)	0.89	(0.46–1.73)
**Highest education among parents**									
	>11 years (ref)	1	1	1	1	1	1	1	1
	8–11 years	1.09	(0.46–2.59)	0.74	(0.51–1.08)	0.84	(0.59–1.19)	0.86	(0.63–1.18)
	<8 years	0.85	(0.10–6.94)	0.55	(0.24–1.26)	0.84	(0.41–1.74)	1.02	(0.53–1.95)
	Unknown	x	x	0.47	(0.10–2.16)	0.79	(0.24–2.60)	1.67	(0.57–4.89)

All: Meet simultaneously the MVPA, screen-time, and sleep duration guidelines; MVPA: Moderate-to-vigorous physical activity; OR: Odds ratio; CI: Confidence Interval; Bold values indicate statistical significance at p<0.05.

The association between sociodemographic factors and meeting the MVPA and sleep duration recommendations measured by accelerometers can be observed in Table 4. Boys were more likely to meet the MVPA recommendation compared to girls (OR = 2.72, 95% CI: 1.42–5.19), but had lower odds of meeting the sleep duration recommendation (OR = 0.56, 95% CI: 0.40–0.78). No statistically significant associations were found for age, SES, number of people in the same household, family structure, and parental education with meeting the MVPA or sleep duration recommendations.

**Table 4:** Sociodemographic correlates of meeting the physical activity and sleep duration guidelines using accelerometer data. (n = 688 adolescents).

	Physical activity		Sleep duration	
	OR	95%CI	OR	95%CI
**Sex**				
Girls (ref)	1	1	1	1
Boys	**2.72**	**(1.42; 5.19)**	**0.56**	**(0.4; 0.78)**
**Age (years)**	0.76	(0.58; 1)	0.91	(0.78; 1.07)
**SES score**	0.99	(0.95; 1.03)	1.00	(0.98; 1.02)
**Number of people in the household**	0.99	(0.77; 1.28)	0.96	(0.84; 1.1)
**Family structure**				
Live with both parents (ref)	1	1	1	1
Single-parent	1.05	(0.53; 2.09)	1.14	(0.78; 1.65)
Does not live with parents	0.7	(0.16; 3.19)	1.22	(0.58; 2.57)
**Highest education among parents**				
>11 years (ref)	1	1	1	1
8–11 years	-	-	1.16	(0.8; 1.69)
<8 years	-	-	0.93	(0.43; 2.04)
Unknown	-	-	1.36	(0.43; 4.33)

OR: Odds ratio; CI: Confidence Interval; The variable of parental education has been omitted from the physical activity regression model, as no participants in the ‘Unknown’ category were inactive, and convergence was not achieved; Bold values indicate statistical significance at p<0.05.

## Discussion

In the present study, we aimed to describe the prevalence and identify sociodemographic factors associated with meeting each and all of the 24-hour movement guidelines in a sample of Brazilian adolescents. Although no sociodemographic factors were associated with meeting all guidelines, some differences were observed for specific recommendations, with boys being more active than girls but with shorter sleep duration. These findings suggest that although no differences are observed for the three recommendations concurrently, specific age and sex subgroups may need different approaches to meet all. These results differ from international studies, where older adolescents [20,21] and girls [18,21] were less likely to meet all guidelines than their counterparts. Overall, the low number of adolescents that met all three guidelines (3.1% of the sample when using self-report and 0.2% when using accelerometer data) suggests that greater efforts are needed to change behaviors. This prevalence is similar to that found in a study with Canadian 10–17 year-olds (3%) [20], but lower than observed in the United States (7%) [21], and in a study with 9–11 year-olds from 12 countries [18] (7.2%). With such a low number of adolescents adhering to the 24-hour movement guidelines, efforts should be made to promote the adoption of healthy 24-hour movement behaviors among adolescents across many regions of the world.

The MVPA recommendation was achieved by 25% of the participants (7.1% when accelerometer data were used) and was lower than screen time and sleep duration recommendations. Low adherence to the MVPA recommendation has been previously reported among adolescents [13], and more specifically among girls [22]. Increasing MVPA levels would be beneficial for the population [41] and healthcare systems [42], and would also increase the proportion of adolescents meeting all 24-hour movement guidelines, as the combination of meeting sleep duration and screen time is most prevalent compared to those including MVPA. Although most interventions in low- and middle-income countries have not been successful [43], new policies and action plans are needed to promote MVPA in this populational subgroup.

Screen time recommendations were achieved by 28% of the participants. Two indicators of screen-time were used in this study, i.e., time watching videos (e.g., movies, series, news), and playing videogames, irrespective if these activities were performed using smartphones, tablets, computers or any other electronic device. This has to be taken into account when comparing the results with other studies, as the metric of screen-time varies with differences in measurement instruments [44]. Large variability has been observed with studies analyzing screen time in Brazilian samples of adolescents, with proportions of non-adherence with the guidelines varying between 9.4% and 68% [44], which may be at least partly attributable to different measures (e.g., only television, or television *and* computer use) and cut-offs used to classify adolescents (e.g., 2 or 4 h/day). Even considering that the evidence for screen-time guidelines is limited [3], the benchmark of 2 h/day is important for research and public health policy [45], and for monitoring this behavior in research. In this study, boys were less likely to meet the screen-time recommendation compared to girls, similar to a study across 12 countries [46]. An inverse relationship was found with age, which is not consistent with current literature [27,47]. One possible explanation for this finding is that as adolescents age, they seem to engage in higher volumes of social media [48] (not currently captured with many screen-time instruments), which may displace time watching videos and playing videogames, giving the wrong impression that time spent on screen-time is diminishing.

Meeting the sleep duration recommendation had the highest adherence compared to MVPA and screen-time, with 41% and 31.7% of participants meeting the guideline when questionnaire and accelerometer data were analyzed, respectively. In a longitudinal study with 11,016 children and adolescents (6–17 years), Faught et al. found that 54.9% of the sample met the sleep duration guidelines in both survey years, while a national-wide study in Canada found that 66.2% of the 22,115 participants met the sleep duration recommendations, a higher proportion compared to MVPA and screen-time recommendations [20]. Although sleep duration was adequate for a higher proportion of adolescents compared to screen-time and MVPA in the present sample of Brazilian adolescents, less than half of the participants slept adequately regularly, which may negatively affect their health. Interventions targeting screen-time may be effective for positively impacting sleep, due to decreasing exposition to blue light [49], and displacing one behavior for the other, which simultaneously helps increase adherence to both and possibly to all 24-hour movement behaviors.

Differences between actigraphy and self-reported measures of sleep in adolescents have been shown to differ greatly [50], which concurs with the difference between the adherence to sleep duration recommendations observed in the present study. Most studies reviewed for the publication of the Canadian 24-hour movement guidelines were from studies that used self-reported sleep [7] and may not be comparable to actigraphy measures of sleep duration. For example, as actigraphy records sleep duration and awakenings with more precision than self-reported instruments, it may be that the equivalent recommended sleep duration when using accelerometers would be lower compared to the value using self-reported instruments. More studies with actigraphy and polysomnography are needed to provide accurate estimates of adequate sleep on health outcomes to support future guidelines.

Parental education, SES, and family structure were not correlated with any of the 24-hour movement behaviors in the present study. These indicators have been associated with the 24-hour movement guidelines in other studies [22], and are useful for identifying underlying inequities in health indicators. However, based on our results, parental education, SES, and family structure may not reflect inequities within the mesoregion where our sample is inserted, but could differ from other cities and states within the country. Although girls could benefit from specific PA interventions, and boys for sleep and screen time interventions, based on our sociodemographic indicators, policies and interventions aiming at the 24-hour movement behaviors should target adolescents from all social backgrounds.

A limitation of the present study is the small size of the sample, which limits the power of inferential statistics (in the case of accelerometer data). Another limitation is the use of self-report instruments to measure habitual behaviors, which are prone to recall limitation and social desirability bias. To address that, we have also included accelerometer measures of sleep duration and MVPA, but it was not possible for screen time, as this behavior can be undertaken using many devices making it challenging to measure with precision. In addition, the cut-off used for classifying MVPA and the algorithm used to identify sleep from accelerometer data were not validated with a wide age group of adolescents, which may limit the accuracy of the estimates for these behaviors in this target population. The main strength of the present study was the use of standard measures to analyze all 24-hour movement behaviors in a sample of adolescents from a middle-income country.

## Conclusions

Approximately 3% of the participants met the MVPA, screen-time, and sleep duration recommendations simultaneously, while this proportion was 0.2% when accelerometer data were used for MVPA and sleep duration. Adherence to the sleep duration recommendation was higher than to the screen-time or MVPA recommendations. Boys were more likely to meet the MVPA recommendations, but less likely to meet sleep duration and scree-time recommendations, and age was positively associated with adhering to the screen-time recommendation. Future policies and interventions should promote adherence to 24-hours movement behaviors in an integrated manner.

## Supporting information

S1 Table Comparison between participants with and without valid accelerometer data (4 days with 16 hours/day, n (%) or mean ±SD). (DOCX)

S2 Table Means of the continuous behavior variables in minutes (n = 688). (DOCX)
