# Supplementary material for: Prevalence and sociodemographic factors associated with meeting the 24-hour movement guidelines in a sample of Brazilian adolescents
Source: PLoS One. 2020 Sep 28;15(9):e0239833. doi: 10.1371/journal.pone.0239833 (PMC7521749; doi:10.1371/journal.pone.0239833)
Supplement: S1 Table — (DOCX) [file pone.0239833.s001.docx]

| **S1 Table.** Comparison between participants with and without valid accelerometer data (4 days with 16 hours/day, n (%) or mean ±SD). | | | | |
| --- | --- | --- | --- | --- |
|  |  | **Self-reported only (n=149)** | **Valid accelerometer data (n= 688)** | **p-value^1^** |
| **Sex** |  |  |  |  |
|  | Girls | 71 (47.7) | 350 (50.9) |  |
|  | Boys | 78 (52.3) | 338 (49.1) | 0.5340 |
| **Age (years)*** | | **16.72 ±1.15** | **16.36 ±1.08** | **0.0003** |
| **SES score [0-100]*** | | 48.95 ±10.35 | 48.92 ±10.00 | 0.9800 |
| **Number of people in the household*** | | 3.64 ±1.17 | 3.83 ±1.21 | 0.0750 |
| **Family structure** | |  |  | 0.0710 |
|  | Live with both parents | 87 (58.4) | 442 (64.2) |  |
|  | Single-parent | 58 (38.9) | 209 (30.4) |  |
|  | Does not live with parents | 4 (2.7) | 37 (5.4) |  |
| **Highest education among parents** | |  |  | 0.6560 |
|  | >11 years | 93 (62.4) | 410 (59.6) |  |
|  | 8-11 years | 49 (32.9) | 226 (32.8) |  |
|  | <8 years | 5 (3.4) | 39 (5.7) |  |
|  | Unknown | 2 (1.3) | 13 (1.9) |  |
| ***Recommendations using self-report*** | |  |  |  |
|  | PA (≥420minutes/week) | **52 (34.9)** | **158 (23.0)** | **0.0300** |
|  | SB (<120minutes/day) | 41 (27.5) | 198 (28.8) | 0.8340 |
|  | Sleep duration (8 ≥ x ≤10h/night) | 53 (35.6) | 292 (42.4) | 0.1460 |
| **Recommendations using accelerometer data** | |  |  |  |
|  | PA (≥60minutes/day) | - | 49 (7.1) |  |
|  | Sleep duration (8 ≥ x ≤10h/night) | - | 218 (31.7) |  |
| *: Mean and standard deviations;  1: *t* tests were used for continuous variables and chi squared tests for categorical data; PA: Physical activity; SB: Sedentary behavior; SES: Socioeconomic score | | | | |
