# Supplementary material for: Prevalence and sociodemographic factors associated with meeting the 24-hour movement guidelines in a sample of Brazilian adolescents
Source: PLoS One. 2020 Sep 28;15(9):e0239833. doi: 10.1371/journal.pone.0239833 (PMC7521749; doi:10.1371/journal.pone.0239833)
Supplement: S2 Table — (DOCX) [file pone.0239833.s002.docx]

| **S2 Table.** Means of the continuous behavior variables in minutes (n=688). | |
| --- | --- |
|  | **Mean (standard deviation)** |
| Accelerometer-measured MVPA | 31.7 (18.2) |
| Self-reported physical activity | 38.68 (46.34) |
| Accelerometer-measured sleep duration | 451.8 (55.8) |
| Self-reported sleep duration | 459.6 (93.0) |
| Screen time (watching videos + playing videogames) | 241.9 (276.9) |
